# An extremely rare case of rapidly growing mediastinal well-differentiated liposarcoma with a sclerosing variant: a case report

**DOI:** 10.1186/s40792-020-00928-4

**Published:** 2020-07-03

**Authors:** Naoya Iwamoto, Yosuke Matsuura, Hironori Ninomiya, Junji Ichinose, Masayuki Nakao, Yuichi Ishikawa, Sakae Okumura, Mingyon Mun

**Affiliations:** 1 Department of Thoracic Surgical Oncology, the Cancer Institute Hospital, Japanese Foundation for Cancer Research, 3-8-31, Ariake, Koto-ku, Tokyo, 135-8550 Japan; 2 Division of Pathology, the Cancer Institute, Japanese Foundation for Cancer Research, Tokyo, Japan

**Keywords:** Well-differentiated liposarcoma, Sclerosing variant, Mediastinal tumor, Rapid growth

## Abstract

**Background:**

Liposarcoma arising from the mediastinum is rare, accounting for less than 1% of mediastinal tumors. Furthermore, a rapidly growing well-differentiated liposarcoma is extremely rare. A well-differentiated liposarcoma is usually considered a low-grade malignancy. However, we present an extremely rare case of a sclerosing variant of well-differentiated liposarcoma that grew rapidly within a year.

**Case presentation:**

A 77-year-old man with a giant mass in the left thoracic cavity was referred to our hospital. This mass measured about 10 cm and occupied the left-sided mediastinum on a chest radiography; however, there was no abnormal finding on the previous year’s chest radiography. Chest-enhanced computed tomography revealed a well-circumscribed 11-cm mass in the left-sided anterior mediastinum. Positron emission tomography showed accumulation of fluorodeoxyglucose uptake in this tumor (maximum standard uptake value = 3.3). The radiological findings of computed tomography and positron emission tomography indicated that this tumor was a benign or low-grade malignancy; therefore, the chest radiographic findings were difficult to explain. To explain this discrepancy and establish the diagnosis, tumor resection was performed via left posterolateral thoracotomy. Intraoperatively, the left phrenic nerve and pericardium were adhered tightly to the tumor, so we resected them. The tumor was well-circumscribed and fibrous; therefore, the initial diagnosis was solitary fibrous tumor. However, based on its histopathological and immunohistochemical patterns, the tumor was diagnosed as a sclerosing variant of well-differentiated liposarcoma. Five years postoperatively, the patient remains alive with no evidence of disease recurrence.

**Conclusions:**

A well-differentiated liposarcoma is usually considered a low-grade malignancy. Nevertheless, the giant tumor in the present case appeared within 1 year. Thus, this was an extremely rare case of a sclerosing variant of well-differentiated liposarcoma with rapid growth.

## Background

Primary mediastinal liposarcoma is rare, accounting for less than 0.5% of total liposarcomas and less than 0.13% of all mediastinal tumors [1]. Furthermore, a rapidly growing well-differentiated liposarcoma (WDLS) is extremely rare. A WDLS is usually considered a low-grade malignancy. Here, we describe an extremely rare case of a rapid growing mediastinal tumor diagnosed as a sclerosing variant of WDLS.

## Case presentation

**Fig. 1:**
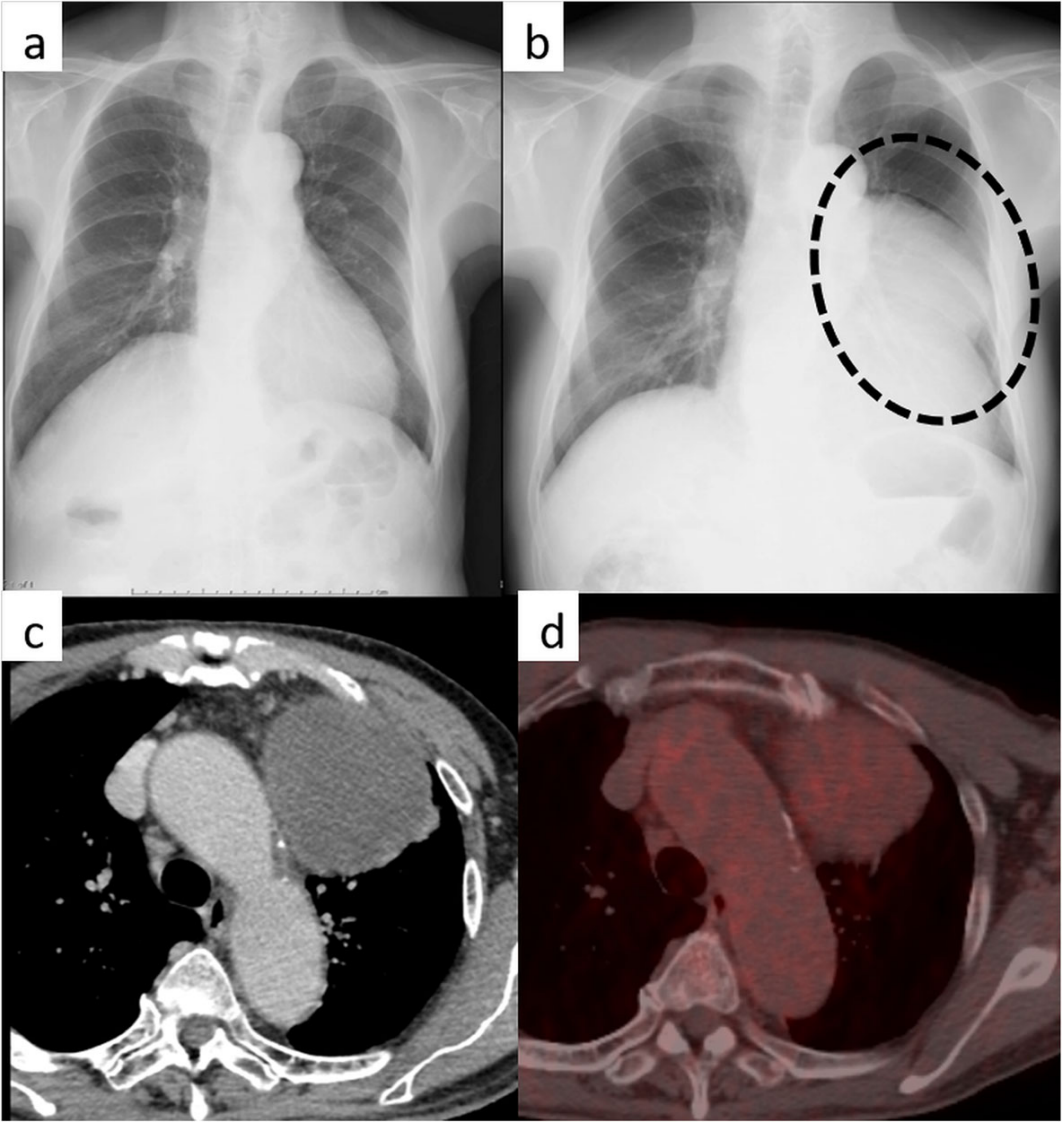
Chest radiography showing the mass (**b**: dotted circle). The mass was not observed a year ago (**a**). Chest-enhanced computed tomography showing an approximately 11-cm mediastinal tumor occupying left internal cavity (**c**). Fluorodeoxyglucose-positron emission tomography showing accumulation in the tumor (maximum standard uptake value = 3.3) (**d**)

A 77-year-old man with a chief complaint of persistent dry cough had an abnormal giant mass on chest radiography (CR) and was referred to our hospital. There were no remarkable findings noted during the physical examination and in blood test results including tumor markers. The giant mass occupied the left-sided mediastinum on CR; however, there was no abnormal finding on the previous year’s CR (Fig. 1a, b). Chest-enhanced computed tomography (CT) revealed a well-circumscribed 11-cm mass in the left-sided anterior mediastinum (Fig. 1c). The mean CT value of the mass was 40 Hounsfield units (range, 20–70), and there were no areas of fatty density. Positron emission tomography-CT showed accumulation of fluorodeoxyglucose uptake in the tumor, with a maximum standard uptake value (SUVmax) of 3.3 (Fig. 1d). We preoperatively diagnosed the benign tumor as a thymoma, solitary fibrous tumor (SFT), or neurogenic tumor, or as a malignant tumor such as thymic cancer, based on the radiological evaluation.

Tumor resection was performed without preoperative biopsy via left posterolateral thoracotomy for diagnosis and treatment. Median sternotomy is considered an alternative surgical approach for mediastinal tumors. In the present case, however, the hilar approach was considered necessary and posterolateral thoracotomy was selected. The tumor was located on the mediastinum and was suspected to invade the left phrenic nerve but not the vagus nerve. The tumor was resected with the left phrenic nerve, thymus, and a part of the pericardium (9.5 × 8.0 cm); therefore, we reconstructed the pericardium using an expanded polytetrafluoroethylene sheet (GORE-TEX®, W. L. Gore & Associates, Co., Ltd., Flagstaff, AZ, USA).

**Fig. 2:**
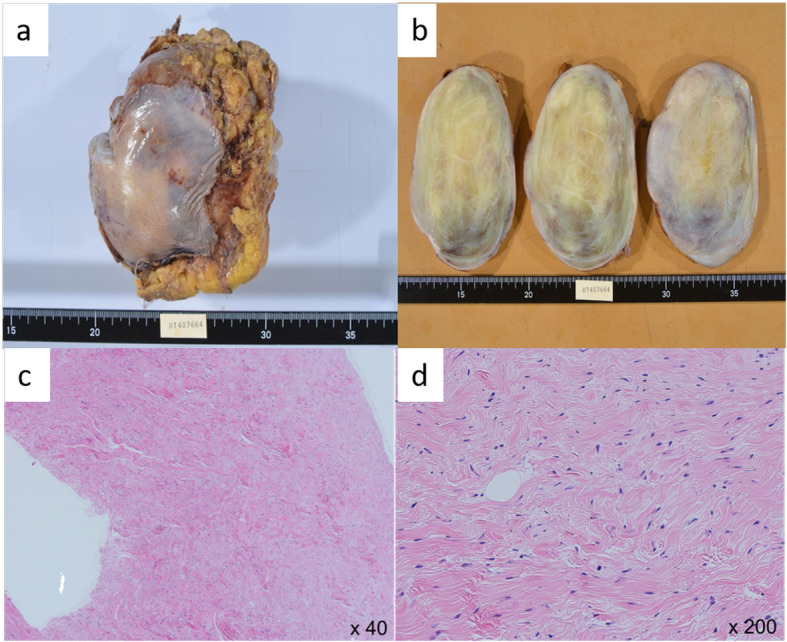
The tumor measures 11.9 × 11.2 × 8.1 cm and is well-demarcated, elastic, hard, and yellowish white with a film (**a**, **b**). Operative rapid pathological findings: the tumor is composed of a rich collagenous fiber and pattern-less spindle cells (**c**, **d**)

**Fig. 3:**
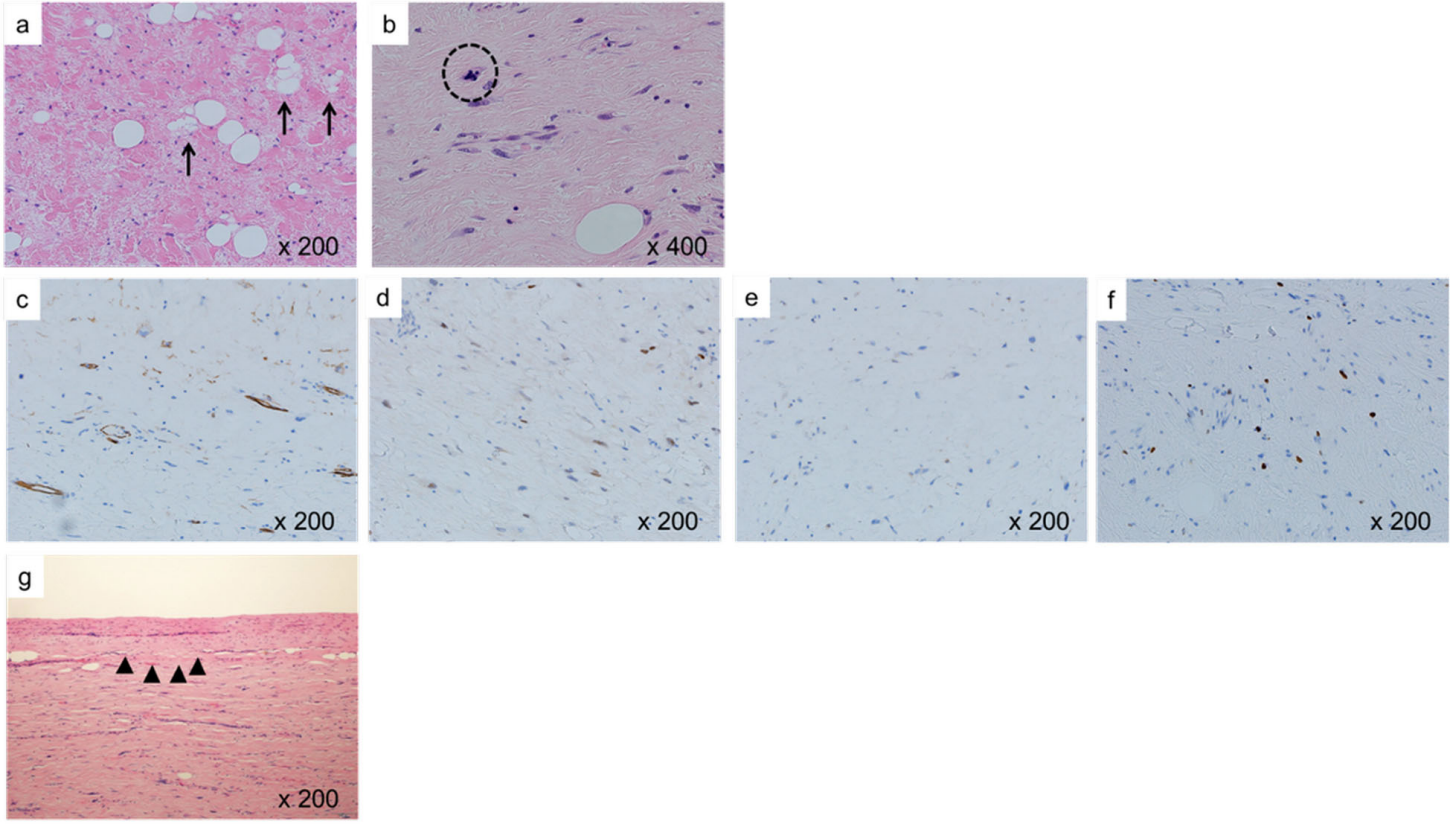
The figure shows a few lipoblasts (**a**: arrows) and mitosis (**b**: dotted circle). Immunohistochemistry shows positivity for CD34 (**c**) and mouse double minute 2 (MDM2) homolog (**d**), and negativity for signal transducers and activator of transcription 6 (STAT6) (**e**). There is positivity for Ki-67, and the MIB-1 index is up to 10% (**f**). The tumor has partially invaded the pericardium (**g**: arrowheads)

Grossly, the tumor measured 11.9 × 11.2 × 8.1 cm, and it was an elastic, well-circumscribed, yellowish, and grayish-white mass (Fig. 2a, b). Histopathologically, the tumor consisted of rich collagenous fiber and pattern-less spindle cells, and these findings suggested SFT (Fig. 2c, d). The tumor, however, comprised a few adipocytes, some of which looked like lipoblasts (Fig. 3a). Moreover, few blood vessels were recognized, and there was no “staghorn vasculature” appearance, which is characteristic of SFT.

Immunohistochemical staining to determine the pathological diagnosis of the tumor was performed using the Leica Bond III automated system (Leica Biosystems, Melbourne, Australia). Necrosis was absent, with up to 10% positivity of Ki-67 (clone, MIB-1; Dako, Glostrup, Denmark; diluted 1:200) (Fig. 3e). Immunohistochemistry showed positivity for CD34 in the endothelium (diluted 1:5; clone, NU-4A1; Nichirei, Tokyo, Japan) and mouse double minute 2 homolog (MDM2) in the nucleus (diluted 1:100; clone, IF2; Invitrogen, Thermo Fisher Scientific, Osaka, Japan) (Fig. 3c,d), while it showed negativity for signal transducers and activator of transcription 6 (STAT6) (diluted 1:200; clone, YE361; Abcam, Cambridge, United Kingdom) (Fig. 3f); these findings were consistent with a diagnosis of WDLS. Furthermore, fluorescence in situ hybridization revealed MDM2 gene amplification.

Based on its histopathological and immunohistochemical patterns, the tumor was diagnosed as a sclerosing variant of WDLS. Sarcoma staging was Fédération Nationale des Centres de Lutte Contre le Cancer (FNCLCC) grade 1 (tumor differentiation = score 1, mitotic count = score 1 [Fig. 3b], tumor necrosis = score 0, total score = 2). The tumor partially invaded the pericardium (Fig. 3 g), but not the phrenic nerve. Although gross complete resection was performed, the pathological margin was positive. Postoperative adjuvant therapies such as chemotherapy and radiotherapy were not performed because of insufficient evidence regarding their efficacy. The patient was alive at 5 years after surgery, with no evidence of disease recurrence.

## Conclusions

Primary liposarcomas arising from the mediastinum are extremely rare. Enzinger and Weiss classified liposarcomas into five histologic subtypes: (1) well-differentiated, (2) myxoid, (3) round cell, (4) dedifferentiated, and (5) pleomorphic [2]. WDLSs are the least aggressive among the five subtypes; previous studies have reported a 5-year survival rate of 87.1% [3] and a recurrence rate of 40–50% [4] for all WDSLs, regardless of their origin. Furthermore, the World Health Organization subdivides WDLS into four morphologic subtypes: (1) lipoma-like (adipocytic), (2) sclerosing, (3) inflammatory, and (4) spindle cell [5]. The sclerosing variant of WDLS is second in frequency only to the lipoma-like form and characterized by the presence of multivacuolated lipoblasts, atypical fibroblasts, primitive mesenchymal cells, and abundant strands of collagen [6]. In the present case, adjuvant therapies were not performed and there was no recurrence. The role of adjuvant chemotherapy or radiotherapy in cases of liposarcoma remains poorly defined [7, 8]. Accordingly, we avoided these treatments and performed regular imaging follow-ups at short intervals. Because of the relative resistance of WDLS to systemic therapy, surgical re-resection has been considered the standard management for recurrent disease. However, patients with rapid recurrence and multifocal disease show poor outcomes, and systemic therapies can be considered in such cases [9].

We found it difficult to diagnose the tumor as WDLS on the basis of the preoperative imaging findings. Gaskin and Helms reported that thickened or nodular septa (generally > 2 mm), associated nonadipose masses, prominent foci showing a high T2 signal, and prominent areas of enhancement in magnetic resonance imaging are all associated with increased risk of WDLS [10]. Although MRI was not performed in the present case, we believe that it may have helped in preoperative differential diagnosis.

To our knowledge, there are few reports about WDLSs with rapid growth. In this case, the SUVmax, FNCLCC sarcoma grading, and MIB-1 index of the tumor were 3.3, grade 1, and up to 10%, respectively. Therefore, radiologically and histopathologically, the tumor was a low-grade malignancy. Nevertheless, this giant tumor appeared within 1 year.

There are several possible reasons for the rapid growth of the tumor. First, there are several reports that low-grade WDLSs grow rapidly secondary to intratumoral hemorrhage; however, there was no such finding in this case. Second, the dedifferentiation of a WDLS relates to rapid growth [11]. A dedifferentiated liposarcoma is defined as a non-fat-forming high-grade sarcoma developing from a WDLS, which is morphologically closely related temporally and spatially to a WDLS, and a non-fat-forming high malignant sarcoma [12]; hence, this histological finding did not match the findings of the present case. Finally, a previous report showed that the sclerosing variant of WDLS with a paucity of fat could mimic high-grade liposarcomas such as a pleomorphic liposarcoma [13]. Furthermore, the sclerosing variant of WDLS may be associated with an increased tendency toward dedifferentiation. In this case, the tumor consisted of rich collagenous fiber and spindle cells with little fatty composition. In summary, the sclerosing variant of WDLS with a paucity of fat is a potentially high-grade liposarcoma that may grow rapidly.

Because the sclerosing variant of WDLS with rapid growth has not been reported, we did not consider this as a possible differential diagnosis preoperatively. Retrospectively, we realized that the sclerosing variant of WDLS with a paucity of fat is a potentially high-grade liposarcoma. These findings indicate that the sclerosing variant of WDLS should be considered in the differential diagnosis for anterior mediastinal lesions with rapid growth; the inherent implications in management and follow-up of these tumors are yet to be determined.

## Data Availability

All related data are included within the article.

## Abbreviations

WDLS: Well-differentiated liposarcoma
CR: Chest radiography
CT: Computed tomography
SUVmax: Maximum standard uptake value
SFT: Solitary fibrous tumor
MDM2: Mouse double minute 2 homolog
STAT6: Signal transducers and activator of transcription 6
FNCLCC: Fédération Nationale des Centres de Lutte Contre le Cancer
